# Computational and experimental methodology for site-matched investigations of the influence of mineral mass fraction and collagen orientation on the axial indentation modulus of lamellar bone^☆^

**DOI:** 10.1016/j.jmbbm.2013.07.004

**Published:** 2013-12

**Authors:** Ewa M. Spiesz, Andreas G. Reisinger, Werner Kaminsky, Paul Roschger, Dieter H. Pahr, Philippe K. Zysset

**Affiliations:** a Institute of Lightweight Design and Structural Biomechanics, Vienna University of Technology, Gusshausstrasse 27–29, A-1040 Vienna, Austria; b Department of Applied Physics, Eindhoven University of Technology, P.O. Box 513, NL-5600 MB Eindhoven, The Netherlands; c Department of Chemistry, University of Washington, Seattle, WA 98195, USA; d Ludwig Boltzmann Institute of Osteology at the Hanusch Hospital of WGKK and AUVA Trauma Centre Meidling, 1st Med. Dept., Hanusch Hospital, Heinrich Collin Str. 30, 1140 Vienna, Austria; e Institute for Surgical Technology & Biomechanics, University of Bern, Stauffacherstrasse 78, CH-3014 Bern, Switzerland

**Keywords:** Collagen fibril orientation, Nanoindentation, Homogenization, Quantitative polarized light microscopy (qPLM), Mineralization, Site-matching

## Abstract

Relationships between mineralization, collagen orientation and indentation modulus were investigated in bone structural units from the mid-shaft of human femora using a site-matched design. Mineral mass fraction, collagen fibril angle and indentation moduli were measured in registered anatomical sites using backscattered electron imaging, polarized light microscopy and nano-indentation, respectively. Theoretical indentation moduli were calculated with a homogenization model from the quantified mineral densities and mean collagen fibril orientations. The average indentation moduli predicted based on local mineralization and collagen fibers arrangement were not significantly different from the average measured experimentally with nanoindentation (*p*=0.9). Surprisingly, no substantial correlation of the measured indentation moduli with tissue mineralization and/or collagen fiber arrangement was found. Nano-porosity, micro-damage, collagen cross-links, non-collagenous proteins or other parameters affect the indentation measurements. Additional testing/simulation methods need to be considered to properly understand the variability of indentation moduli, beyond the mineralization and collagen arrangement in bone structural units.

## Highlights

• Site-matched assessment of nanoindentation modulus, mineral mass fraction and collagen fibers orientation in human cortical bone sections.
• Comparison of experimental nanoindentation modulus with its computed equivalent based on the site-matched morphological data.
• While mean experimental and computed nanoindentation moduli match well, their variations exhibit very weak correlations.
• Considering factors like nano-porosity and damage may be necessary to understand variability of lamellar stiffness of bone structural units.
• This is not in conflict with the well known anisotropy associated with the rotated plywood model at the sublamellar scale.

## Graphical abstract

**Figure:**
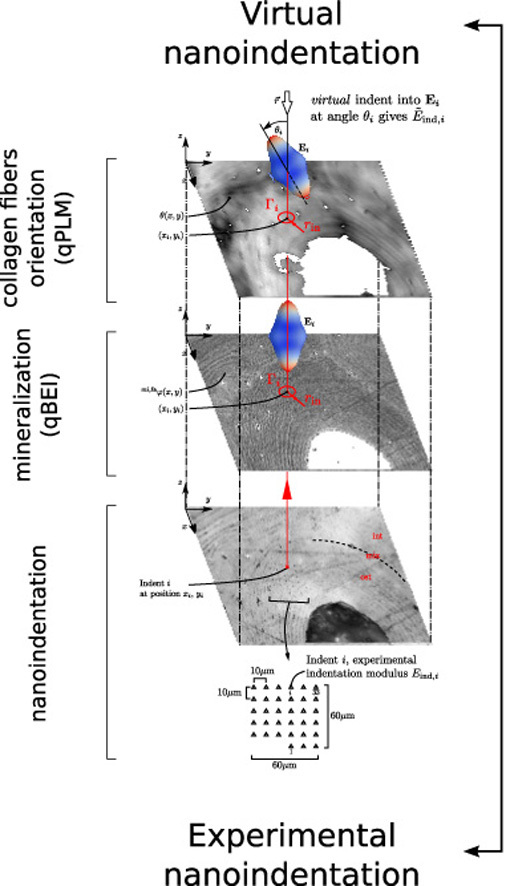

## 1 Introduction

The impact of composition and structural arrangement on mechanical properties of cortical bone tissue has been investigated intensively in the past decades (Fratzl and Weinkamer, 2007; Currey, 2002; Weiner et al., 1999).

At the macroscopic level, the influence of mineralization on the stiffness of cortical bone has been identified in early work (Vose and Kubala, 1959; Currey, 1969), especially when considering a broad range of species (Currey, 2002). The anisotropic nature of the elastic properties of compact bone was recognized in the same period and attributed to collagen organization (Dempster and Liddicoat, 1952; Reilly and Burstein, 1975).

At the scale of bone structural units (BSU), three different alignment patterns were distinguished in collagen fibrils, and according to the either dark, bright or intermediate appearance in polarized light microscopy (PLM), osteons are expected to follow either longitudinal, transverse or alternating collagen orientations (Ascenzi et al., 2003; Ascenzi and Lomovtsev, 2006). Ascenzi et al. identified the osteon as the mechanical unit of compact bone (Ascenzi and Bonucci, 1967) and reported a significant role of mineralization and collagen arrangement on their tensile and compressive properties (Ascenzi and Bonucci, 1967, 1968). The BSU was also recognized as the mechanical unit of bone tissue in nanoindentation studies and the substantial variability in indentation modulus measured along the axis of osteons was attributed to the their distinct mineralization and collagen alignment pattern (Zysset et al., 1999; Boivin et al., 2008; Manjubala et al., 2009; Fan et al., 2002).

At the microscopic level, a single lamella of collagen arrangement exhibits a complex irregularly rotated plywood pattern (Giraud-Guille, 1998; Weiner et al., 1999; Wagermaier et al., 2006). Nanoindentation experiments by Gupta et al. (2006b) using smaller than usual final indentation depth of ∼137nm revealed an intra-lamellar modulation of mechanical properties, with significantly stiffer thick lamellar subregions, as compared to the thin sublamellar region. The anisotropic stiffness of this sophisticated structure, measured by nanoindentation (Franzoso and Zysset, 2009), was well predicted by a unit cell homogenization approach (Reisinger et al., 2011a). Uniaxial mineralized collagen fibril arrays have recently been examined by Reisinger et al. (2010) who concluded that among the various factors affecting the composite stiffness, mineral density should have a dominant role.

Recent experimental evidence using ultrasound microscopy or nanoindentation confirmed the influence of collagen orientation, while on the other hand the measurements failed to show any strong correlation between the amount of mineral and stiffness of bone tissue (Raum et al., 2006; Hengsberger et al., 2002a; Zebaze et al., 2011). In contrast, other authors show a dependency of stiffness on mineral content (Boivin et al., 2008; Manjubala et al., 2009; Fratzl-Zelman et al., 2009; Gupta et al., 2006b).

However, the structure–function relationships were previously not quantified on a local site-matched basis. Accordingly, the following hypothesis is formulated in this work: “axial stiffness of lamellar bone measured by nanoindentation (in dry condition) is fully determined by mineral mass fraction and mean out-of-plane collagen fibril angle”.

Testing the hypothesis requires correlating the mineral density and mean out-of-plane collagen fibril angle with indentation moduli in a site-matched fashion. By means of statistical analysis, we intend to verify if a theoretical indentation modulus calculated from a homogenization model, based only on experimental mineral fraction and collagen fibril orientation as input parameters, predicts the observed variability in experimental axial indentation modulus.

## 2 Materials and methods

### 2.1 Sample preparation

**Fig. 1:**
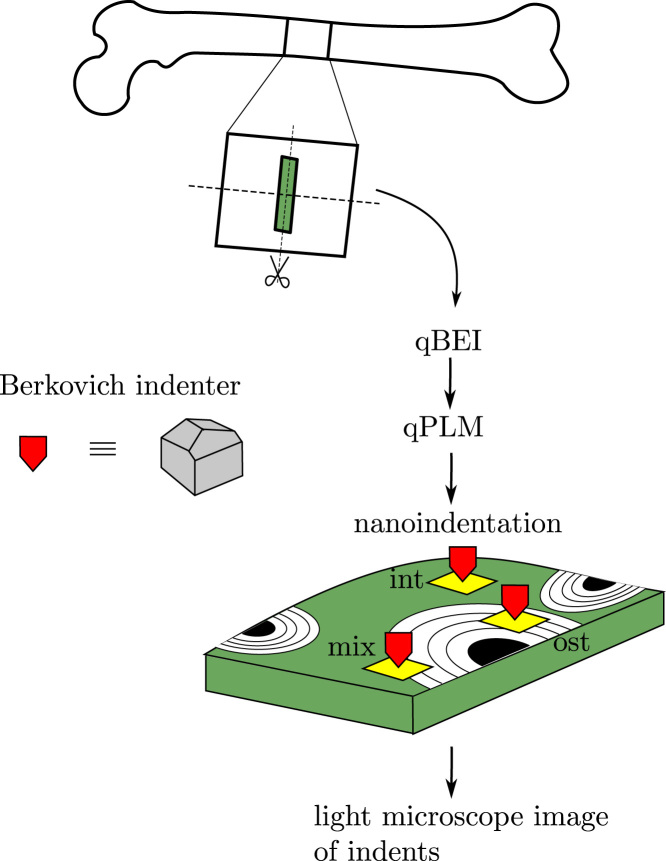
Study design and sample preparation. The slices were imaged by qBEI and qPLM and then indented in three regions (ost)eonal, (int)erstitial, (mix)ed. Light microscope images of the indented sites were taken.

Human femurs from two donors (a male of 68 and a female of 89 years old) were dissected and kept frozen in −20° until the day of sample preparation. The donors were free from bone related pathologies and the samples were obtained via an informed consent. Slices of approximately 10 mm were cut from the mid-shaft of the femurs using a low-speed precision diamond band saw (Exakt Vertriebs GmbH, Norderstedt, Germany) under constant water irrigation, see Fig. 1. Out of those two samples slices of approximately 1 mm were cut using the same machine.

Such prepared slices were washed in a soap solution in de-ionized water for 7 min in an ultrasonic bath, followed by a 7 min bath in a 2% bleach solution and than washed under running de-ionized water (Franzoso and Zysset, 2009; Wolfram et al., 2010a). After the cleaning procedure, the samples were dried in room temperature for 12 h. Parallel slices of ∼1mm thickness were glued to glass slides using Loctite 420 glue (Henkel Loctite Adhesives Ltd., UK) and polished with a semi-automatic polishing machine Logitech PM5 (Logitech Limited, Glasgow, UK) until obtaining 30−60μm sample thickness.

### 2.2 Quantitative backscattered electron imaging (qBEI)

Quantitative backscattered electron images (qBEI) were captured using a digital scanning electron microscope with a four-quadrant semiconductor backscattered electron detector (DSM 962, Zeiss, Germany), at working distance 15 mm (Roschger et al., 1995, 1998). Probe current was adjusted to 110±0.4 pA and electron beam energy used was 20 keV. Sample surfaces were coated with carbon for qBEI analyses (Fratzl-Zelman et al., 2009; Roschger et al., 1998). This layer was removed before further quantitative polarized light microscopy and nanoindentation measurements using an alcohol solution. Images with 50× and 400× magnification were captured resulting in a scanned area of approximately 2.0×2.5 mm and 250×315μm, respectively (Spiesz et al., 2012a, 2012b).

At 400× nominal magnification 1 pixel corresponded to a sample surface area of $0.5\times 0.5\;\mu m^{2}$, with the penetration depth of 1–1.5μm. The penetration depth has been previously estimated using Monte Carlo simulations of electron scattering (Goldstein et al., 2002; Howell and Boyde, 1994, 2003). Under the above described experimental settings of qBEI imaging, the overwhelming part of electrons backscattered from the sample surface and detected by the detector were coming from a sample depth (escape depth/sampling volume) of 1–1.5μm. However, the actual lateral resolutions achieved with the current set-up appeared to be much higher – down to the submicron range. Lamellar fibril arrangement patterns and osteocytes canaliculi cross sections have been resolved using the same qBEI settings (Roschger et al., 1993).

The images served as maps allowing recognition of the zones where mineralization was assessed for subsequent indentation. Concentration of calcium (weight % calcium, ψca,fa) was determined (Roschger et al., 1998). Both meso- and visible microporosity were thresholded. The inorganic phase consisting of calcium phosphate was idealized as hydroxyapatite ${\mathrm{Ca}}_{10}{\left({\mathrm{PO}}_{4}\right)}_{6}{\left(\mathrm{OH}\right)}_{2}$. The calcium wt.% (ψca,fa) was converted to hydroxyapatite mass fraction (ψca,fa – mass fraction of the mineral in the fibril array) using stoichiometric relationships as in Roschger et al. (1998) and Manjubala et al. (2009). The volume fraction of the mineral in the fibril array φmi,fa accounting for nano-porosity was calculated using an empirical relationship between φmi,fa and the ultra-structural mass density from Fritsch and Hellmich (2007).

**Fig. 2:**
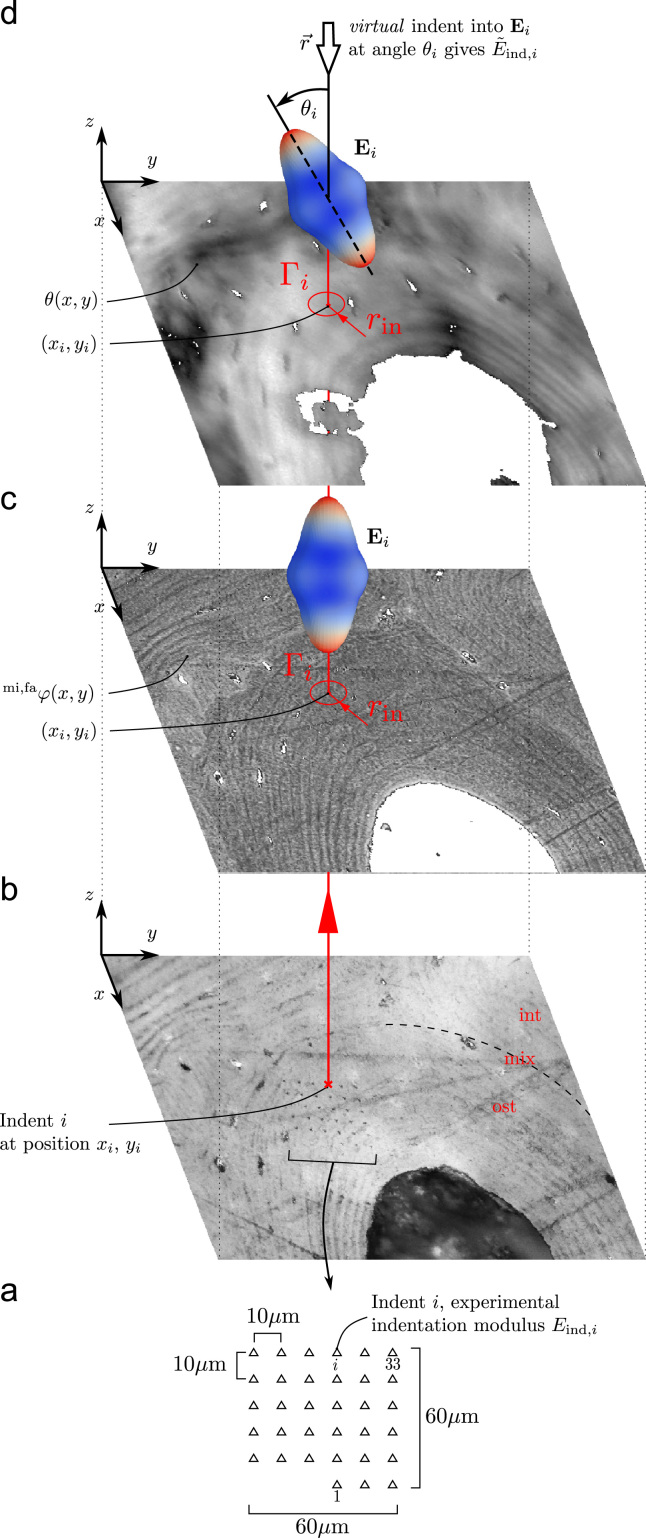
Site-matched image analysis. (a) 29 indent patterns are distributed on osteons (ost), the interstitial zones (int) and not clearly identifiable regions (mix). (b) The position $\left(x_{i},\;y_{i}\right)$ of each indent *i* is assessed by light microscope images. (c) Calibrated qBEI images of the indentation zone, displaying the tissue mineral volume fraction φmi,fa(x,y), are registered to the light microscope images. The average φmi,fa in the indent–surface interaction zone $\Gamma_{i}$ is used to model the local bone tissue stiffness tensor $E_{i}$. (d) Calibrated qPLM images of the indentation zone, displaying the local fibril angle θ(x,y) relative to the sample surface normal, are registered to the qBEI and light microscope images. The average fibril angle in the indent–surface interaction zone $\Gamma_{i}$ is used to model an appropriately oriented indent into the material $E_{i}$ yielding ${\overset{˜}{E}}_{\mathrm{ind},i}$.

(1) φmi,fa=Bψmi,faψmi,fa−AρHA

with *A*=0.59 ml/g and B=−0.75. The density of hydroxyapatite $\rho_{\mathrm{HA}}$ was set to 3.16 g/cm^3^ (de With et al., 1981). As a result, images of osteonal bone, showing the local degree of mineralization φmi,fa(x,y) as volume fraction were obtained (Fig. 2c).

### 2.3 Quantitative polarized light microscopy (qPLM)

A polarized light microscopy technique used before in a qualitative fashion by Martin and Ishida (1989), Boyde and Riggs (1990), Bromage et al. (2003) and Skedros et al. (2009), was recently calibrated for a quantitative use on thin bone sections by Spiesz et al. (2011a) and Spiesz (2011). The out-of-plane collagen arrangement angle θ (the angle that collagen fibrils form with the normal to the specimen surface) in a parallel fibered mineralized turkey leg tendon (MTLT) was calibrated to the linear birefringence of MTLT cut at various angles with respect to the longitudinal axis. The calibration curve was normalized to sample thickness and wavelength of the probing light to enable a universally applicable quantitative assessment (Spiesz et al., 2011a). The angle θ for bone regions of interest represents an out-of-plane collagen fibril angle averaged over that region and sample thickness. The system used consisted of a microscope for polarized light, interference filter and broadband quarter-wave retarder to produce incident circular polarized light, as well as an image multiplexer (Quadview, MAG Biosystems, BioVision Technologies Inc., PA, USA) and a CCD camera (Kaminsky et al., 2007; Spiesz et al., 2011a). The qPLM observations result in images with a gray-scale coded collagen fibril angle θ(x,y) computed for each pixel (Fig. 2d).

### 2.4 Nanoindentation

The acquired qBEI images served as maps for the selection of the regions of interest for indentation. In each sample section 5–30 qBEI images were taken at 400×, defining the regions of interest for indentation. Indentations were performed in dry condition using a TriboIndenter (Hysitron Inc., MN, USA). A Berkovich tip was used and measurements were conducted in displacement control until 500 nm depth using a loading/unloading rate of 40 nm/s and a holding time of 30 s (Reisinger et al., 2011b). Patterns of 33 indents were placed within the regions of interest (Fig. 2a). The pattern size was approximately 60×60μm.

Three types of regions of interest were defined: (ost)eonal – within an osteon, (int)erstitial – in the interstitial tissue, (mix)ed – partially within an osteon and partially within the interstitial tissue, as shown in (Fig. 1).

The patterns were scanned with the Berkovich tip after indentation in the scanning probe microscopy mode of the TriboIndenter in order to detect any abnormalities in the surface nearby the indents or problems with surface detection resulting in a corrupt indent size. The local surface roughness estimated from the scanning probe microscopy mode of the TriboIndenter in areas of $5\times 5\;\mu m^{2}$ was ranging in 50–100 nm. Therefore the indentation depth to surface roughness ratio was 10:1–5:1 – in the range not affecting the measured indentation stiffness (Donnelly et al., 2006).

Additionally, each pattern was imaged with light microscopy in order to localize the indentation sites in the qBEI and qPLM images.

The following 2-step indent filtering procedure was applied in order to remove the corrupt indents:

1. Indents that could not be clearly identified on surface images or that were placed in or nearby pores or cracks were excluded.
2. Indents, which load–displacement curve contained irregularities from the regular shape were discarded.

### 2.5 Site-matched assessment of $E_{\mathrm{ind}}$, φmi,fa and θ

To allow a site-matched assessment of measured indentation modulus $E_{\mathrm{ind}}$, degree of mineralization φmi,fa and fibril orientation θ, the obtained light microscopy images, qBEI images and qPLM images were registered (Fig. 2). The semi-automatic registration procedure involved two steps. First the three images were registered visually. Then an automatic optimization procedure involving minimization of a normed difference between parts of the images via small relative rigid body movements was employed.

For an indentation modulus $E_{\mathrm{ind},i}$ at position $\left(x_{i},\;y_{i}\right)$ on the light microscope image, a corresponding mineral volume fraction φimi,fa and fibril orientation angle $\theta_{i}$ were extracted from the images as the average over the indentation area:

(2) φimi,fa=1Ain∫Γiφmi,fa(x,y)dΓθi=1Ain∫Γiθ(x,y)dΓ

The field data φmi,fa(x,y) and θ(x,y) are averaged on a circular area $\Gamma_{i}$ with the radius $r_{\mathrm{in}}$, centered at the respective indent location $\left(x_{i},\;y_{i}\right)$ (Fig. 2c and d). This circle represents the interaction zone between indenter tip and sample surface and holds a diameter of seven times the indentation depth $2r_{\mathrm{in}}=7\times 500\;\mathrm{nm}=3500\;\mathrm{nm}$ (Hengsberger et al., 2002b). Its area is therefore $A_{\mathrm{in}}=\pi r_{\mathrm{in}}^{2}$.

### 2.6 The modeled indentation modulus ${\overset{˜}{E}}_{\mathrm{ind}}$

For each measured indentation modulus $E_{\mathrm{ind},i}$, a corresponding *virtual* indentation modulus ${\overset{˜}{E}}_{\mathrm{ind},i}$ is now estimated that is based on the local mineral volume fraction φimi,fa and fibril angle $\theta_{i}$. This is achieved by the following two-step procedure.

#### 2.6.1 Fibril-array model

First, the local degree of mineralization φimi,fa is used in a micro-mechanical material model of the fibril-array to calculate the stiffness tensor $S_{i}$ of the local bone matter (Fig. 2c).

When modeling the tissue elastic properties at an individual indentation location, the model has to reflect the bone microstructure at the length scale of the indentation influence zone $\Gamma_{i}$. The zone's diameter of $2\times r_{\mathrm{in}}=3.5\;\mu m$ is around half the width of an average osteonal lamella in human bone which is approximately 5–7μm (Rho et al., 1998). On this sub-lamellar length scale, the fibril organization is assumed to be rather unidirectional, array-like. Parallel mineralized collagen fibrils are embedded in a mineralized extra-fibrillar matrix (Lees et al., 1994).

**Fig. 3:**
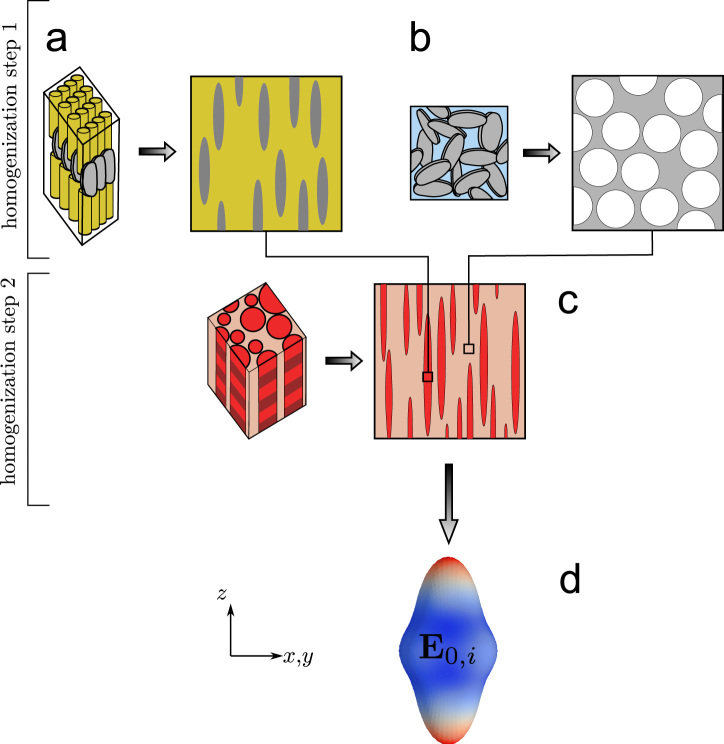
Mean field model representations of bone structure and multiscale work flow: (a) the fibril structure is modeled as a composite with prolate isotropic mineral spheroids unidirectionally embedded in an isotropic collagen matrix; (b) the extra-fibrillar mineral–protein network is modeled as a mineral foam with spherical voids; (c) the fibril-array is built of highly elongated prolate spheroids of fibril material, embedded in extra-fibrillar matrix material; (d) the output is a transverse isotropic stiffness tensor of the fibril-array.

In this context, a multiscale micromechanical fibril-array model may be used (Reisinger et al., 2010; Spiesz, 2011; Spiesz et al., 2011b). It applies mean field methods to model mineralized fibrils, the extra-fibrillar matrix and the resulting fibril-array (Fig. 3). The transverse isotropic elastic properties of the fibril-array are computed as a function of degree of mineralization, mineral distribution between fibrils and extra-fibrillar matrix, collagen stiffness and fibril volume fraction.

In this work, all input parameters except the degree of mineralization are set to constant values listed in (Table 1) that are supposed to conform with average human lamellar bone. The local transverse isotropic stiffness tensor $S_{i}$ of the bone tissue within the deformed zone of an indent *i* is then calculated as a function of local mineralization φimi,fa only.

#### 2.6.2 Virtual indentation

In a second step, the indentation modulus ${\overset{˜}{E}}_{\mathrm{ind},i}$ for a virtual indent into the modeled material is estimated using the theory of Swadener and Pharr (2001). The appropriate direction for this virtual indent is defined by $\theta_{i}$, the angle between the fibril- and the experimental indentation direction (Fig. 2d):

(3) $${\overset{˜}{E}}_{\mathrm{ind},i}={\overset{˜}{E}}_{\mathrm{ind}}(r(\theta_{i}),S_{i})\;\mathrm{with}\;r(\theta )=[\begin{array}{c} 0\\ \sin(\theta )\\ \cos(\theta ) \end{array}]$$

with ${\overset{˜}{E}}_{\mathrm{ind}}$ being a function returning the indentation modulus of a virtual indent into a material of stiffness $S_{i}$ in arbitrary direction defined by the vector r described in the material coordinate system (Reisinger et al., 2011b).

An indentation modulus ${\overset{˜}{E}}_{\mathrm{ind},i}^{0}={\overset{˜}{E}}_{\mathrm{ind}}(r(0),S_{i})$ that neglects the influence of the fibril orientation and that is just based on the local mineralization is gained by holding $\theta_{i}$ constant at 0° (indentation in the axial direction of the fibrils).

### 2.7 Statistics

Comparison between the osteonal and interstitial regions was performed using the Tukey multiple comparison of means test (Crawley, 2005). The level of significance was set to α=0.05.

Comparison between the measured and computed indentation moduli means was performed using a non-parametric Wilcoxon rank test and *t*-test. The Wilcoxon test is more conservative than the *t*-test: if a difference is significant under a Wilcoxon test it would be even more significant under the *t*-test (Crawley, 2005).

The correlation between the measured and the modeled indentation moduli was determined by calculating the squared Pearson product-moment correlation coefficient *r*^2^ of a linear dependence.

Preliminary ANOVA analyses identified the donor as non-significant factor. Thus the donor was not included as factor in the final statistics. The requirements of homoscedacity on the data are fulfilled: the response values are approximately normally distributed, independent and the variances in the groups are similar.

## 3 Results

Twenty nine indentation patterns, each with up to 33 valid indents were distributed on several samples (Fig. 2a). In total, 883 measurement sites $\Gamma_{i}$ were included in the statistical evaluation (out of 957 performed). Out of this total amount 438 measurement sites were located in the osteonal tissue, 290 in the interstitial one and the rest of the sites included a mixture of both tissue types. The site-matched regions were assessed with qBEI, qPLM, nanoindentation and virtual nanoindentation of which the results are discussed below.

### 3.1 qBEI results

**Fig. 4:**
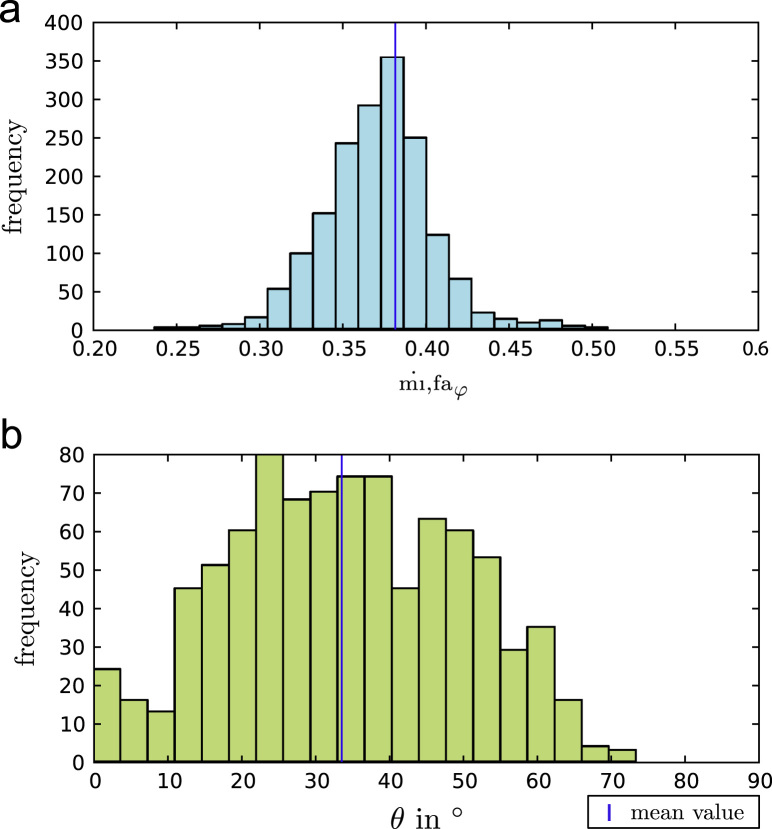
(a) Histogram of the estimated local mineral volume fraction φimi,fa (*n*=883). (b) Histogram of the measured local fibril- or collagen angle $\theta_{i}$ relative to the sample surface (*n*=883).

The mean and standard deviation of the measured calcium weight fraction were ψca,fa=0.25±0.02. The resulting estimation of mineral volume fraction φmi,fa was 0.38±0.02 and its distribution is shown in (Fig. 4a). Interstitial tissue showed higher mineralization with φmi,fa=0.39±0.03 as compared to the osteonal tissue φmi,fa=0.38±0.02. The difference was highly significant with p<0.0001.

### 3.2 qPLM results

At the same sites, the mean fibril or collagen angle $\theta_{i}$ relative to the sample surface was measured. The mean and standard deviation of the measured out-of-plane collagen angle were 32.3°±14.7° with angles between 0° and 73.2°. The distribution of θ is shown in (Fig. 4b). The difference in θ was not significant between the osteonal ($\overset{¯}{\theta }=31.9\pm 15.5{}^{\circ}$) and interstitial ($\overset{¯}{\theta }=32.9\pm 13.2{}^{\circ}$) regions (*p*=0.96).

### 3.3 Experimental and virtual indentation results

After applying the filter procedure described in Section 2.4, 883 indents were considered valid. The mean and standard deviation of the measured indentation moduli $E_{\mathrm{ind}}$ were 23.99±5.12 GPa. The corresponding values of $E_{\mathrm{ind}}$ in the osteonal and interstitial tissues were 24.22±4.80 GPa and 23.66±5.56 GPa, respectively. The differences between osteonal and interstitial zones were not significant (*p*=0.63).

The mean and standard deviation of the predicted indentation moduli ${\overset{˜}{E}}_{\mathrm{ind}}$ were 24.77±2.66 GPa. The corresponding values of ${\overset{˜}{E}}_{\mathrm{ind}}$ in the osteonal and interstitial tissues were 24.42±2.47 GPa and 25.29±2.84 GPa, respectively.

The means of the measured $E_{\mathrm{ind}}$ and the modeled indentation moduli ${\overset{˜}{E}}_{\mathrm{ind}}$ were not significantly different with *p*=0.9 (Wilcoxon test).

The average predicted indentation modulus when the fibril angle is fixed at θ=0° (not accounting for the measured collagen angle) is higher at 27.54±2.07 GPa, as this indents are performed parallel to the stiff fibril direction.

### 3.4 Correlation

Four correlation analyses were performed between the measured indentation moduli $E_{\mathrm{ind}}$, see (Table 2), and the mineral fraction φmi,fa, the fibril angle θ, the computed ${\overset{˜}{E}}_{\mathrm{ind}}^{0}$ and ${\overset{˜}{E}}_{\mathrm{ind}}$.

**Fig. 5:**
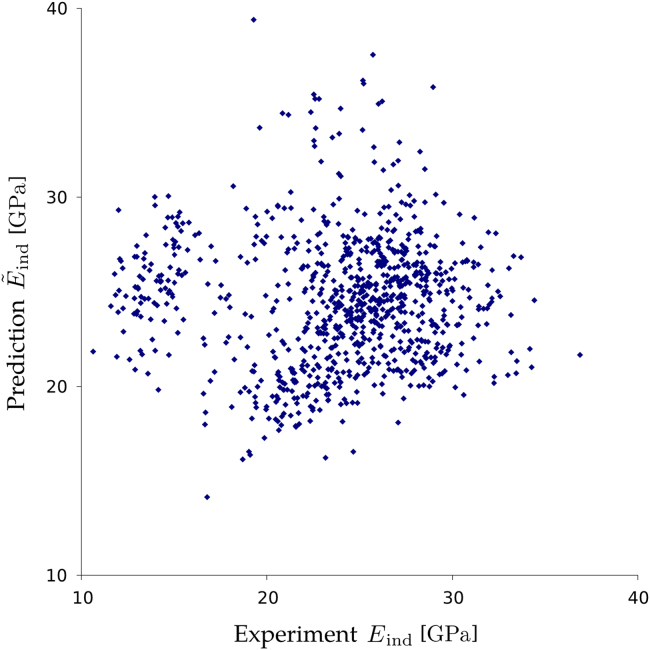
Lack of correlation between the measured indentation modulus $E_{\mathrm{ind}}$ and the prediction ${\overset{˜}{E}}_{\mathrm{ind}}$, which is based on the local tissue mineralization as well as on the local fibril orientation. Total number of points: *n*=883.

Surprisingly, no substantial correlations were found between those variables. The lack of correlation between the measured indentation moduli and the one predicted using the other measured variables (φmi,fa, θ) is shown in (Fig. 5).

**Fig. 6:**
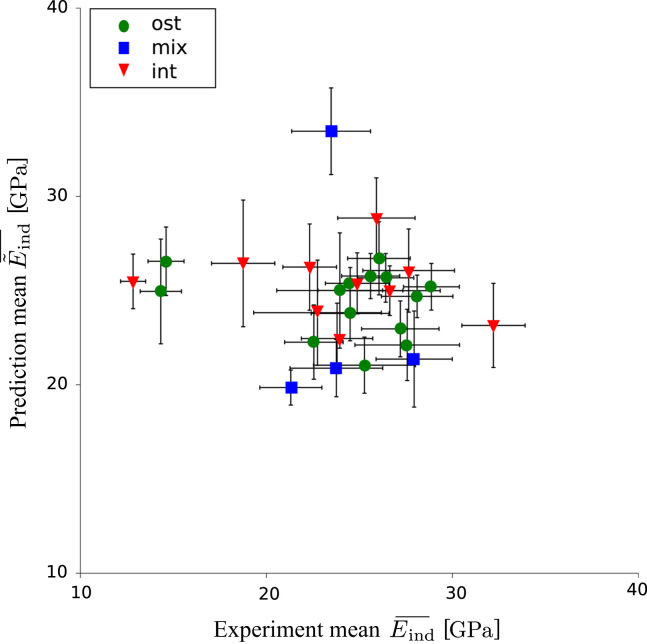
Lack of correlation between the measured mean indentation modulus $\overset{¯}{E_{\mathrm{ind}}}$ and the mean predictions $\overset{¯}{{\overset{˜}{E}}_{\mathrm{ind}}}$, which is based on the local tissue mineralization as well as on the local fibril orientation within one indentation pattern. The symbols indicate different regions where the indent patterns were placed (ost)eonal, (mix)ed, (int)erstitial. The error bars indicate the intra-pattern standard deviations of $\overset{¯}{E_{\mathrm{ind}}}$ and $\overset{¯}{{\overset{˜}{E}}_{\mathrm{ind}}}$. Total number of points: *n*=29.

The trends were similar when looking at averaged values measured in different sites with the distinction of tissue type (osteonal, interstitial, mixed). Fig. 6 shows the comparison of the averaged measured indentation moduli ($\overset{¯}{E_{\mathrm{ind}}}$) and the modeled ones ($\overset{¯}{{\overset{˜}{E}}_{\mathrm{ind}}}$). In none of the different regions, a perceptible trend can be identified.

## 4 Discussion

This work tests the hypothesis that the variability of axial lamellar bone stiffness measured by nanoindentation can be determined by mineral mass fraction and mean collagen orientation. Multiple experimental as well as numerical methods were utilized. Before approving or rejecting this hypothesis, the results are discussed below.

The average measured calcium weight fraction of 0.25±0.02 in the tested samples is slightly higher than the average reported in the literature. In Roschger et al. (2003) the normal trabecular bone showed a calcium mass fraction of 0.23, similar to the results obtained by Fratzl-Zelman et al. (2009). The significant difference in mineralization between the osteonal and interstitial regions (*p*=0.0016) is attributed to the local age of the tissue. In the ongoing mineralization process, the younger osteons are less mineralized compared to the older interstitial regions (Zysset et al., 1999; Boivin and Meunier, 2002; Roschger et al., 2008).

Some previous work has been done on evaluation of the effects of the average arrangement of the mineralized collagen fibrils on stiffness (Ramasamy and Akkus, 2007; Bakbak et al., 2011; Granke et al., 2013). The average out-of-plane collagen angle θ measured in this study was 32.3°±14.7°, which is in agreement with the literature. An acoustic microscopy study performed by Turner et al. (1995) showed the principle direction of collagen averaged over an approximately 60μm thick bone sections to be about 30°. Similar average arrangement of the fibrils was indirectly measured by Wagermaier et al. (2006) with a micro-beam X-ray diffraction method, with the difference that the arrangement of the mineral crystallites, not collagen fibrils, was measured. This result can be compared to the present study with the assumption that the mineral follows the collagenous matrix in the average arrangement. This is likely the case for the mineral placed within the collagenous fibrils, but the arrangement of the hydroxyapatite crystallites may be more random within the extra-fibrillar matrix.

The θ-distribution of the current study is compatible with the investigations of Ascenzi et al. (2003) who reported two classes of osteons – a *dark* class with a mainly axial fibril alignment and a *bright* class containing mainly fibrils aligned at ∼45°. However, when looking at the distribution of θ in (Fig. 4) it is striking that some few values reach 73° but no transversely oriented fibrils at ∼90° were observed at this resolution. This fact is not in line with the earlier investigations of Ascenzi and Bonucci (1967, 1968) who proposed a class of *transverse* osteons with a main fibril orientation of θ=90°.

In Reisinger et al. (2011b) the major principal stiffness direction relative to the osteon axis was evaluated by nanoindentation. The average angle was reported to be around 10°. Assuming that the stiffness alignment is reflected in the mean fibril alignment, these results would be lower than the present qPLM data.

The average indentation modulus $E_{\mathrm{ind}}$ measured here was 23.99±5.12 GPa, which can be considered within the range of moduli measured by nanoindentation with similar final indentation depth seen in the literature. Franzoso and Zysset (2009) measured an average of 22.3±2.2 GPa with nanoindentation of osteons extracted from similar anatomical location. Reisinger et al. (2011b) measured 20.5–27.6 GPa, depending on the angular cut of an osteon (from transverse to axial direction of indentation with respect to the osteon axis). Lower indentation moduli were measured with microindentation performed until a final indentation depth of 2.5μm by Wolfram et al. (2010a, 2010b) ranging in indentation moduli 10.65±0.16 GPa till 15.00±0.14 GPa depending on the origin and direction in which the trabecular bone was indented. A certain indentation depth effect – a decrease of the measured indentation modulus with increasing indentation sampling volume, was observed previously in a variety of materials, also mineralized tissues (Swadener and Pharr, 2001; Zhang et al., 2008; Voyiadjis and Peters, 2010; Spiesz et al., 2012a).

The average indentation modulus estimated with the fibril array model ($\overset{¯}{{\overset{˜}{E}}_{\mathrm{ind}}}=24.77\pm 2.66\;\mathrm{GPa}$) was not significantly different from the measured values (*p*=0.9), which suggests that the level of lamellar bone stiffness is well predicted.

In the present study including osteons with a rather narrow range of mean mineralization, we saw no correlation between mineralization and the measured indentation stiffness ($r^{2}=0.00$), see (Table 2), even weaker than the ones previously shown in the literature (Raum et al., 2006; Boivin et al., 2008; Follet et al., 2004; Zebaze et al., 2011). Hengsberger et al. (2002a) reported a *r*^2^ of 0.42 for a 86 year old donor and, a *r*^2^ of 0.00 for a 30 year old donor suggesting an age dependence. Some encouraging results on the dependence of tissue mechanical properties on the collagen phase arrangement have been presented (Ramasamy and Akkus, 2007), mixed with some discouraging ones (Bakbak et al., 2011).

The expected improvement of correlation after considering the collagen arrangement was very weak, see (Table 2). This suggests that there are other important factors influencing the local tissue stiffness fluctuation. The hypothesis that the variability of the lamellar bone stiffness, measured by nanoindentation, can be expressed by mineral mass fraction and mean collagen orientation, was therefore *rejected*, at least for osteons with a narrow range of mineralization and the experimental conditions used in the study. This finding does not challenge the previously presented dependence of bone elastic properties on mineralization and collagen orientation at different hierarchical material levels (Hengsberger et al., 2002a; Gupta et al., 2006a; Roschger et al., 2010; Oyen et al., 2008), but signifies that the mean mineralization and the mean collagen orientation among bone structural units are generally relatively homogeneous and that other factors may influence the variation in local tissue stiffness.

Possible limitations that may influence the obtained correlations are now to be discussed.

We tried to explain the high variability of the measured indentation modulus for a specific mineralization in a small auxiliary test, based on the Monte Carlo method. The starting point was the highest observed span of indentation moduli $E_{\mathrm{ind}}$, which occurred at φmi,fa=0.34, where indentation moduli between 10 GPa and 35 GPa were measured (difference of 25 GPa). We investigated if this variability could be explained by the fibril-array model when applying reasonable changes on the input parameters of (Table 1) and the fibril orientation θ. For answering this question, the mineral quota of total mineral in the fibrils and the fibril volume fraction in the fibril-array, were both varied between 0.25 and 0.75. The fibril orientation angle θ was varied between 0° and 90°. In the course of the Monte Carlo method, this variation was performed randomly and 10,000 sets of input parameters were created. The resulting predicted indentation modulus ${\overset{˜}{E}}_{\mathrm{ind}}$ ranged from 5 GPa to 21 GPa (difference of 16 GPa). This indicates, that the high local fluctuation of measured stiffness cannot be fully explained by neither of the three investigated parameters used in this setting.

One of the possible reasons for the low correlation might be the fact that the method of mineral content evaluation used here is nonvolumetric. The used qBEI technique relates the mass fraction of calcium to the intensity of the electrons backscattered with high energy from a bone section (Roschger et al., 1998). Depending on the architecture of the tissue and its porosity, possibly different tissue volumes are tested pixel by pixel. If the nano-porosity (not resolved by the technique) varies within an image, this introduces errors in the mineral volume fraction estimation. As the mineral volume fraction is the key input parameter for mechanical models, the resulting properties are distorted.

Additionally, the fact that the range of mineral volume fraction of the analyzed osteons was rather limited may contribute to the low correlation found between indentation modulus and mineralization.

Beside the qBEI issue, undefined nano-porosity can influence the nanoindentation measurements. Pores in the indent–surface interaction zone soften the structure and lead to lower indentation moduli. A site-matched assessment of nano-porosity would be needed in order to overcome this problem.

In a two-case study by Hengsberger et al. (2002a), samples from an elderly donor showed a significantly lower indentation modulus than the ones from a younger donor despite its higher mineralization. This finding converges with the outcome of the present study and might be attributed to micro-damage, collagen cross-linking or noncollagenous proteins, three factors that were not accounted for in our mean field homogenization model. The excessive accumulation of micro-damage was recently shown to affect the indentation modulus (Dall'Ara et al., 2012; Schwiedrzik and Zysset, 2013). The donors in the present study were also rather old and may be prone to inherent micro-damage.

As qBEI and nanoindentation operate on the tissue surface, the surface related alterations due to the tissue preparation have to be considered. As shown in Roschger et al. (1993), repeated wetting and drying introduces ultra-cracks in the tissue. This increase of porosity might lead also to a local softening. However, it seems improbable that such a degradation is approximately homogeneous within bone structural units and at the same time independent of the extent of mineralization. Additionally, a number of investigations suggested the potential role of collagen cross-linking and non-collagenous proteins in the mechanical integrity of bone matrix, e.g. (Saito and Marumo , 2010; Thurner et al., 2010; Paschalis et al., 2011). The extent of their respective roles in bone indentation stiffness in human bone structural units remains to be quantified.

The use of regular patterns for selection of the zones of indentation imply that a random fluctuation depending if the zone falls in the center or in the edge of a lamella appears consistently in all the measurements. In principle, all the measurement were performed at the same location at the same length scale and this should therefore be insensitive to the above fluctuation. However, the out-of-plane collagen angle evaluated here is an average over the specimen thickness (approximately 30–60μm). This means that different sublamellae or even lamellae are averaged, depending on the sample cut. In contrast, the indentation moduli describe the stiffness of the top surface of a sample (depth of approximately 500 nm). Therefore the discrepancies between the measured and the predicted indentation moduli may arise from variability of collagen angles within the 30–60μm sample thickness. This effect that did not prove critical in our validation study on unidirectional mineralized turkey tendon (Spiesz et al., 2011a) may jeopardize the lateral resolution of the qPLM measurements in lamellar bone.

Finally, the planar registration procedure of the different images has also a finite accuracy of a fraction of a micron that may shift the zones of interest with respect to each other and weakens the sought correspondence.

The uniaxial structure considered in the homogenization model could be seen as another limitation, as the material within an indentation site (3.5μm in diameter) may not be oriented uniaxially. According to the observations of Weiner et al. (1999), a lamella is formed by 5 uniaxial sub-lamellae with various orientations. Approximately 80% of the sub-lamellae showed a mean out-of-plane collagen angle within 0° and 30°. This non-uniaxial arrangement was also demonstrated by Wagermaier et al. (2006) using SAXS and Gupta et al. (2006b) using nanoindentation with a smaller indentation depth, but a recent study by Varga et al. (2013) showed more homogeneous fibril arrangement within lamellae.

## 5 Conclusion

In this study, a method for a site-matched correspondence of the indentation modulus of bone to its mineral mass fraction and mean collagen fibril orientation is proposed. Even though the measurements and simulation have several limitations, the results suggest that the variation of indentation modulus among lamellar bone structural units cannot be explained by mineral mass fraction and mean collagen orientation, which points towards the role of other factors such as nano-porosity, damage, collagen cross-linking and noncollagenous proteins. Finally, there was no evidence for a class of transverse osteons in the obtained fibril angle distribution for the considered femoral diaphyses.

## Figures and Tables

**Table 1:** Set of input parameters for the fibril-array model of Reisinger et al. (2010).

Input parameter	Value	Reference
Collagen Young's modulus	5 GPa	Cusack and Miller (1979)
Collagen Poisson ratio	0.3	–
Mineral Young's modulus	110.5 GPa	Yao et al. (2007)
Mineral Poisson ratio	0.28	Yao et al. (2007)
Mineral platelet aspect ratio in fibril	14	Akkus (2005)
Void aspect ratio in extra-fibrillar matrix	1	–
Fibril aspect ratio in fibril-array	100	–
Fibril volume fraction in fibril-array	0.53	Fritsch and Hellmich (2007)
Mineral quota of total mineral in fibril	0.25	Lees et al. (1994)

**Table 2:** Squared Pearson correlation coefficient *r*^2^ for the dependency of the measured indentation moduli $E_{\mathrm{ind}}$ (last row) on: (1) mineralization φmi,fa; (2) fibril angle θ; (3) computed indentation modulus ${\overset{˜}{E}}_{\mathrm{ind}}^{0}$ disregarding the fibril orientation and (4) computed indentation modulus ${\overset{˜}{E}}_{\mathrm{ind}}$ including the local fibril orientation. Additionally, the respective means and standard deviations are listed.

Var	Mean	*r*^2^ value
φmi,fa	0.38±0.02	0.0000
θ	32.3°±14.7	0.0035
${\overset{˜}{E}}_{\mathrm{ind}}^{0}$ (comp)	27.54±2.07 GPa	0.0001
${\overset{˜}{E}}_{\mathrm{ind}}$ (comp)	24.77±2.66 GPa	0.0005

$E_{\mathrm{ind}}$ (exp)	23.99±5.12 GPa	–
